# Supplementary material for: Flood insurance is a driver of population growth in European floodplains
Source: Nat Commun. 2023 Nov 18;14:7483. doi: 10.1038/s41467-023-43229-8 (PMC10657371; doi:10.1038/s41467-023-43229-8)
Supplement: Supplementary file 1 — Supplementary Information [file 41467_2023_43229_MOESM1_ESM.pdf]

Supplementary Material

Model calibration and sensitivity analysis

In this section we discuss how the model is calibrated and present the sensitivity of the simulation to several key variables, including various scenarios of climate- and socio-economic change (as RCP-SSP combinations), different amenity values, and an extension of the analysis to 2080. Variables obtained from existing models, i.e. GLOFRIS and earlier versions of DIFI, are calibrated or validated based on existing data, as discussed in subsequent articles [1, 2]. Earlier studies with the DIFI model also show that model output is coherently responsive to input parameter changes [2, 3].

The DIFI-model extension designed for this study is calibrated using findings from [4]. This study observed the growth of the population in floodplains relative to population growth outside floodplains, on country-level, and for the period 2000-2015. For calibration purposes we executed the model developed in this study over the same period and generated model output in a comparable format. To do this, population growth statistics on NUTS3-level were obtained from Eurostat, and flood damage data for the specific period was obtained by extrapolating data from GLOFRIS. This enables us to simulate the change in the proportion of the population exposed to floods from 2000-2015, which we are able to aggregate to country-level in order to compare and calibrate the model to empirical findings in [4]. The amenity value of river proximity is the variable used to calibrate the expected utility functions (Equations 2 and 3 in the manuscript). Because [4] does not show results for some European countries, we performed the calibration on countries that did show results, including France, the UK, Germany, the Czech Republic, Poland, and Romania. Status-quo insurance arrangements were used to compare model output to results in [4], meaning flat-rate insurance premiums for France and Romania, and risk-based premiums for all remaining countries. The results of calibration show a slight overestimation from results in [4] of, on average over the considered countries, 18%. This overestimation is largely driven by our projection for the UK, which shows a 50% overestimation when considering the current insurance system. However, the flood insurance system in the UK was not yet firmly established in the period 2000-2015 [5], which may explain at least part of the overestimation. Our projection for the UK without insurance availability shows only a slight overestimation of 10%. Apart from the UK, our result show roughly similar patterns as in [4]. In particular, countries that show relatively high floodplain population growth there (i.e. Germany, Romania) also show higher floodplain population growth compared to other countries in our analysis.

The degree to which the simulation is sensitive to amenity values is assessed and shown in Figure 1. More specifically, this figure shows results in the same format as Figure 1, but with modified amenity values. To test the model sensitivity to amenity values of river proximity, for this analysis we raised the value of amenities, while enhancing the amount by which amenities decay with distance from the

river. These amenity values are, therefore, more aligned with findings in studies such as [6]. The most notable changes that arise due to the different amenity values occur in the scenario without insurance availability. Comparing Panels B in Figure 1 in the manuscript and in 1 below it can be seen that floodplain population growth increases when amenity values close to rivers are higher. Closer inspection of the data reveals that this increase counts for 30% of all regions in Panel B, while it is 20% in Panel C. The reason that the scenario with insurance availability is less sensitive to higher amenity value is that this value is less important to determine whether a floodplains settlement location is preferable over the high ground. When flood risk is covered, and particularly when this coverage is cheap, households will quickly prefer living in close proximity to a river when there are positive amenities. On the other hand, when flood risk is not covered, or coverage is expensive, households may only choose the floodplain settlement location over the high ground when the amenity value of a river is considerable. Whereas flood risk outweighing amenities may often be a motivation for choosing the high ground when flood insurance is not available, when flood risk becomes less relevant in the settlement decision (i.e. particularly under insurance with a flat-rate premium structure) households may only be discouraged from settling close to a river for other reasons. These reasons may include the unaffordability of higher housing prices close to rivers, but also that some regions are ill suited to have a high population density due to remoteness. In our simulation we included an affordability condition, where a household is excluded from settling in the floodplain if the amenity value of it's floodplain settlement location ($A_{j,i,t}$) is larger than it's wealth endowment ($W_{j,i,t}$). Also, we maintain a cap on the population density in a region, which is sensitive to the distance of a region to urban centres. Data on the maximum population density of a region is provided by the 2UP-model, and explained in [7].

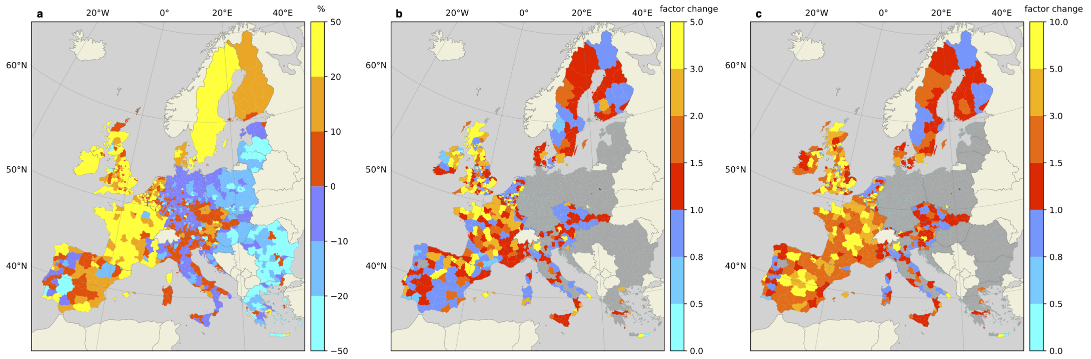

Fig. 1: Sensitivity of population growth projections developed in this study to amenity values. This figure shows results where amenity values are higher and decline more rapidly over distance compared to the results shown in Figure 1 in the manuscript. **(a)** Projected population development in floodplains from 2010 to 2050 in percentages using the baseline method under SSP2. Blue shades indicate a decline in floodplain populations, while red to yellow shades indicate an increase. **(b)** the difference from the baseline projection when considering environmental (dis)amenities of floodplains and household-level DRR. **(c)** the difference with the baseline-projection when considering insurance availability in addition to the determinants included in Panel **b**. In Panels **b** and **c**, blue shades indicate a lower floodplain population compared to the baseline approach, while red to yellow shades indicate a higher population projection. For regions depicted in grey, our method is not applicable due to declining populations across these regions.

Besides amenity values, the chosen climate change- (RCP) and socio-economic (SSP) scenarios are important exogenous variables that impact the settlement location decision in this study. Climate change impacts future flood hazard, which makes it important to test how flood risk may develop differently depending on the level of climate change and considering population change in floodplains. Population development in a NUTS3-region impacts the potential exposure growth within floodplains, where a higher population growth means more households make the decision whether to settle in- or outside floodplains. To test the model sensitivity to climate change and population parameters, we apply different RCP-SSP combinations to the analysis introduced in this study. Figure 2 presents the growth of populations in floodplains from 2010 to 2050 in the regular format for three different scenarios of climate- and socio-economic change (RCP4.5-SSP1; RCP4.5-SSP2; RCP8.5-SSP5). It can be seen that under SSP5 (Panel G) many more NUTS3-regions show population growth in floodplains than under SSPs 1 and 2 (Panels A and D). As expected, this projection is aligned with the increasing overall population under this scenario in Figure 4 in the main text.

The difference in model output between SSP1 and SSP2 (Panels A to F) is limited, which is largely because differences in the general population growth between these two socio-economic scenarios is modest. Panels G to I show more significant deviations with the above plots. A distinct difference is that more NUTS3-regions are included in the analysis, particularly in Germany, Italy and Hungary. The reason for this is that populations in these regions are projected to grow only in the SSP5-scenario. Whereas deviations in population growth when considering flood risk, adaptation options, and amenities (Panel

H) shows a similar pattern to regions in other scenarios (Panels B and E), the extent of these deviations is larger under the RCP8.5-SSP5 scenario (i.e. bright blue and yellow colors occur more frequently in Panel H than in panels B and E). One reason for this projection is that overall population growth in European regions is considerably higher under SSP5, as can be seen in Panel G, which means more households face the settlement location choice in our simulation, raising the potential for deviation with the baseline. The main driver of floodplain population growth remains flood risk, which is also more severe under the RCP8.5 scenario in many parts of Europe [8]. Introducing status-quo insurance coverage (Panel I) means that the negative impact of flood risk on the expected utility of floodplain settlement is considerably reduced, particularly when insurance coverage is cheap (i.e. France and Spain), causing more households to opt for the floodplain amenities. Again, a higher number of households facing the settlement location choice under SSP5 allows for more outstanding results compared to the previous scenarios.

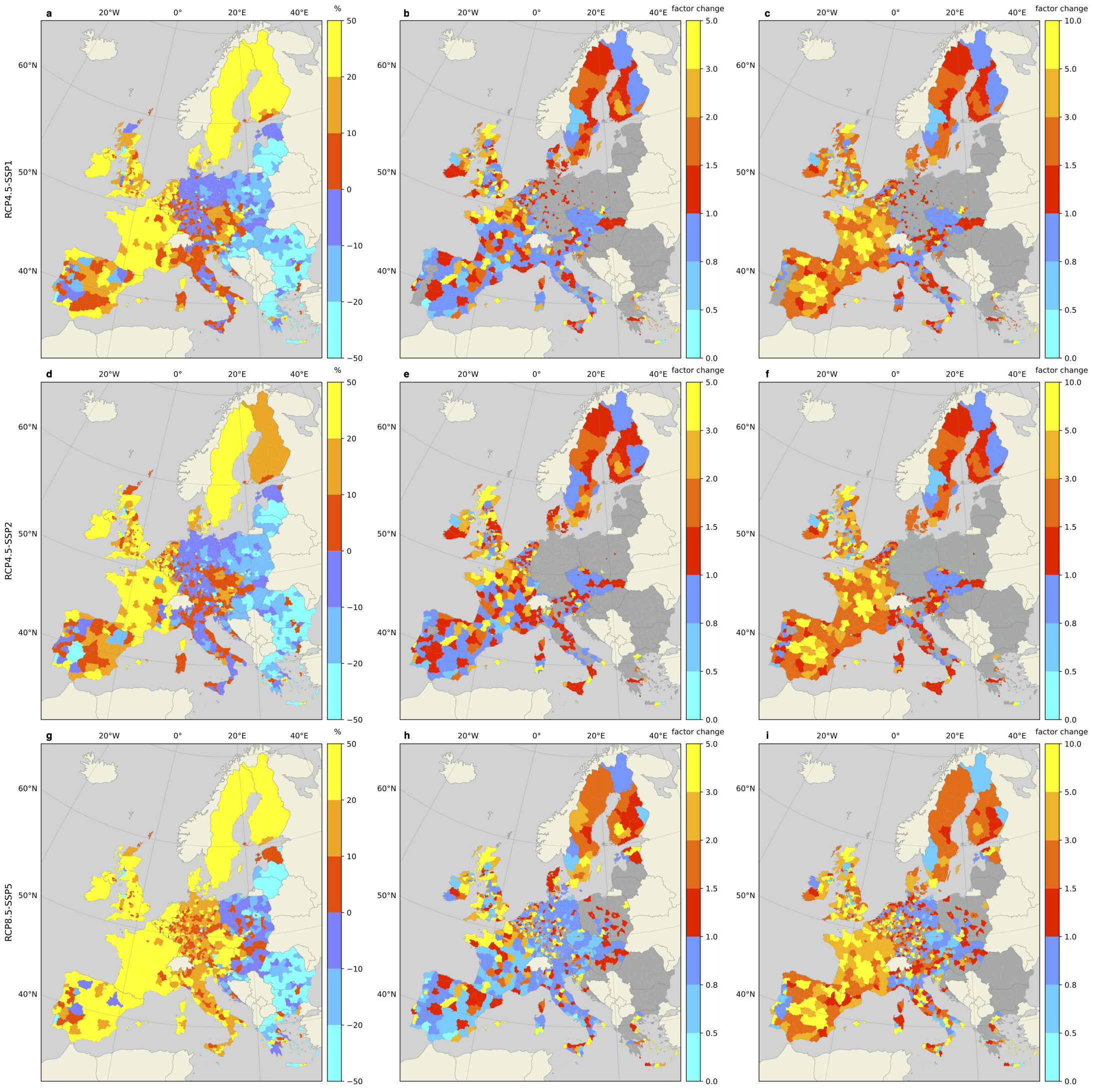

Fig. 2: Population projections in the standard format of this study, but for three distinct scenarios of climate- and socio-economic change (RCP-SSP combinations). The top row (Panels **a-c**) show results for RCP4.5-SSP1; the middle row (Panels **d-f**) for RCP4.5-SSP2, which are the results shown in Figure 1; and the bottom row (Panels **g-i**) show results for RCP8.5-SSP5).

Climate- and socio-economic scenarios differ in their impact on population growth in floodplains. Higher flood exposure, due to population growth in floodplains, increases flood risk. To interpret the results regarding changing flood risk due to exposure developments presented in this study, it is useful to compare the results in Figure 2 of the main text with the growth in flood risk caused by a higher degree of climate change. Panel A of Figure 3 below presents the increase in EAD under RCP8.5-SSP5, while Panel B shows the difference between Panel A and the projection of EAD under RCP4.5-SSP2 with insurance availability. Panel B, therefore, compares climate change and insurance availability as a driver of flood risk. The prominence of the colour blue indicates that in many regions exposure growth caused

by insurance is a stronger driver of flood risk than more severe climate change. This result is particularly evident in countries with flat insurance premiums, and most notably in France. Which of the drivers is most prominent in countries where premiums are risk-based is less obvious, although an average value of -1.5 indicates that, overall, also in these countries insurance is a stronger driver of flood risk.

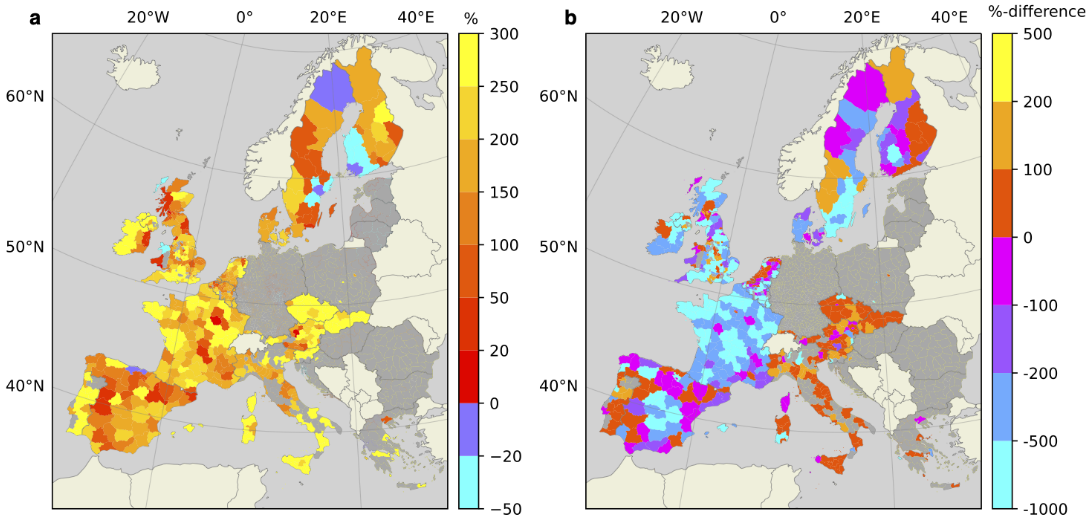

Fig. 3: Comparing the impacts of climate change and insurance availability on flood risk. (**a**) The percentage change in EAD from 2010 to 2050 under RCP8.5-SSP5 and under the baseline population scenario. (**b**) The percentage difference between panel **a** and the change in EAD under RCP4.5-SSP2 considering insurance availability (Panel **c** of Figure 1). The prominence of red to yellow colours in panel **a** indicates that EAD increases for most regions under RCP8.5-SSP5. The prominence of blue, purple, and pink in panel **b** indicates that the increase in EAD over the same period is higher under RCP4.5-SSP2 when considering insurance availability.

So far, we examined population and flood risk development until 2050. Assessing policy impacts, such as flood insurance policy, 2050 is a more realistic objective than end-of-century assessments. However, as impacts of climate change are likely to become more severe in the second half of the century [9], extending our analysis to include such impacts of climate change may show to what extent households potentially adapt in terms of their settlement location with respect to riverine flood risk, as well as emphasize the importance of flood insurance policy. Figures 4 and 5 present floodplain population development and consequent changes in EAD under RCP4.5-SSP2 from 2010 to 2080. First of all, we observe a continuation of population changes within floodplains in the baseline scenario (Panel A of Figure 4), where population growth in several Western- and Northern European countries becomes higher, while in other regions it declines further. With respect to the baseline projection, our simulations until 2080 (Panels B and C) seem to result in equal or higher projections of population growth in floodplains for almost all regions, compared to the analysis until 2050 (Figure 1 in the main text). This indicates that, despite almost consistent increases in flood risk under the baseline scenario after 2050, households are still more likely to opt for the floodplain when deciding where to settle.

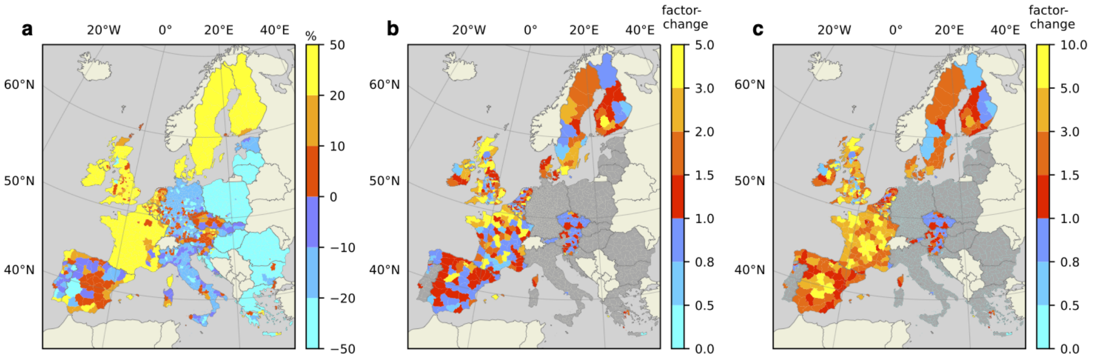

Fig. 4: Change in population living in floodplains from 2010-2080. **(a)** Projected population development in floodplains from 2010 to 2080 in percentages using the baseline method under SSP2. Blue shades indicate a decline in floodplain populations, while red to yellow shades indicate an increase. **(b)** the difference from the baseline projection when considering environmental (dis)amenities of floodplains and household-level DRR. **(c)** the difference with the baseline-projection when considering insurance availability in addition to the determinants included in Panel **b**. In Panels **b** and **c**, blue shades indicate a lower floodplain population compared to the baseline approach, while red to yellow shades indicate a higher population projection. For regions depicted in grey, our method is not applicable due to declining populations across these regions.

Due to higher climate- and socio-economic change, EAD is projected to increase to a higher degree towards 2080 compared to projections until 2050 (see Panel A of Figure 5). Moreover, the difference in EAD with the baseline projection is consistent with simulations of floodplain population growth in Figure 4. The difference in floodplain population growth between status-quo and risk-based insurance pricing (Panels C and D) is larger in the analysis until 2080 (compared to Figure 2 in the main text), which stresses the importance of the design of flood insurance systems.

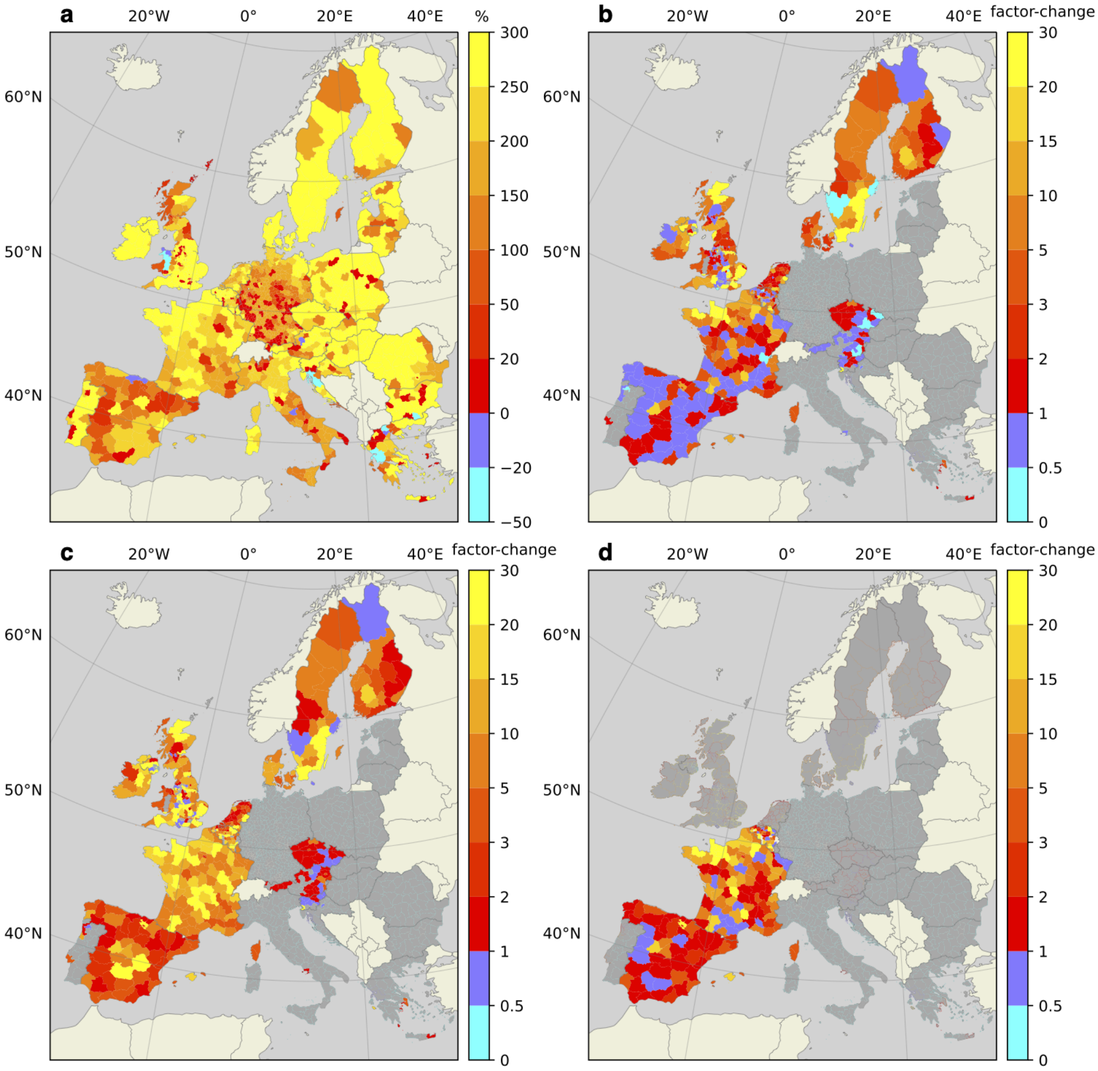

Fig. 5: Change in riverine flood risk from 2010-2080. (**a**) The projected growth of EAD from 2010 to 2080 in percentages, under the baseline population model. Blue shades indicate a decline in EAD, while red to yellow shades indicate an increase. (**b**) The factor change with respect to Panel **a** when applying our population growth model that respects environmental (dis)amenities of floodplains and household-level adaptation. (**c-d**) The factor change with respect to Panel **a** when also considering the availability of flood insurance, where Panel **c** considers status-quo insurance arrangements, while Panel **d** displays a change to risk-based insurance premiums in countries that currently have flat-rate premiums. Blue shades in Panels **b-d** indicate lower EAD compared to Panel **a**, while red to yellow shades indicate a higher projection. For regions depicted in grey, our method is not applicable due to declining populations across these regions. Panel **d** also displays countries with risk-based premiums in grey.

References

[1] Ward, P. J. *et al.* A global framework for future costs and benefits of river-flood protection in urban areas. *Nature Climate Change* **7** (9), 642–646 (2017). http://www.nature.com/articles/nclimate3350. 10.1038/nclimate3350 .

[2] Hudson, P., Botzen, W. W. & Aerts, J. C. Flood insurance arrangements in the European Union for future flood risk under climate and socioeconomic change. *Global Environmental Change* **58**, 101966 (2019). https://linkinghub.elsevier.com/retrieve/pii/S0959378018306022. 10.1016/j.gloenvcha.2019.101966 .

[3] Tesselaar, M., Botzen, W. W., Robinson, P. J., Aerts, J. C. & Zhou, F. Charity hazard and the flood insurance protection gap: An EU scale assessment under climate change. *Ecological Economics* **193**, 107289 (2022). https://linkinghub.elsevier.com/retrieve/pii/S0921800921003487. 10.1016/j.ecolecon.2021.107289 .

[4] Tellman, B. *et al.* Satellite imaging reveals increased proportion of population exposed to floods. *Nature* **596** (7870), 80–86 (2021). https://www.nature.com/articles/s41586-021-03695-w. 10.1038/s41586-021-03695-w .

[5] Surminski, S. & Eldridge, J. Flood insurance in England - an assessment of the current and newly proposed insurance scheme in the context of rising flood risk. *Journal of Flood Risk Management* **10** (4), 415–435 (2017). https://onlinelibrary.wiley.com/doi/10.1111/jfr3.12127. 10.1111/jfr3.12127 .

[6] Tapsuwan, S., MacDonald, D. H., King, D. & Poudyal, N. A combined site proximity and recreation index approach to value natural amenities: An example from a natural resource management region of Murray-Darling Basin. *Journal of Environmental Management* **94** (1), 69–77 (2012). https://linkinghub.elsevier.com/retrieve/pii/S0301479711002532. 10.1016/j.jenvman.2011.07.003 .

[7] van Huijstee, J., van Bemmel, B., Bouwman, A. & van Rijn, F. Towards an Urban Preview: Modelling Future Urban Growth with 2UP. Tech. Rep. 3255, PBL Netherlands Environmental Assessment Agency, the Hague (2018). https://www.pbl.nl/sites/default/files/downloads/pbl-2018-Towards-an-urban-preview$_3$255*.pdf*.

[8] Winsemius, H. C. *et al.* Global drivers of future river flood risk. *Nature Climate Change* **6** (4), 381–385 (2016). http://www.nature.com/articles/nclimate2893. 10.1038/nclimate2893 .

[9] Hirabayashi, Y. *et al.* Global flood risk under climate change. *Nature Climate Change* **3** (9), 816–821 (2013). http://www.nature.com/articles/nclimate1911. 10.1038/nclimate1911 .
